# Competition for the Senn Medal

**Published:** 1897-08

**Authors:** J. McFadden Gaston

**Affiliations:** Chairman; 1½ Edgewood Ave., Atlanta, Ga.


### COMPETITION FOR THE SENN MEDAL.

Pursuant to a resolution adopted by the Section of Surgery and Anatomy of the American Medical Association, June 4, 1897, I have been appointed by the chairman, Dr. Reginald H. Sayre, as chairman of the committee charged with the awarding of the Senn Medal for 1898. The other members of the committee are Drs. H. O. Walker, of Detroit, Mich., and S. H. Weeks, of Portland, Me.

1. A gold medal of suitable design is to be conferred upon the member of the American Medical Association who shall present the best essay upon some surgical subject.

2. This medal will be known as the Nicholas Senn Prize Medal.

3. The award shall be made under the following conditions: (*a*) The name of the author of each competing essay shall be enclosed in a sealed envelope, bearing a suitable motto or device, the essay itself bearing the same motto or device. The title of the successful essay and the motto or device to be read at the meeting at which the award is made, and the corresponding envelope to be then and there opened and the name of the successful author announced. (*b*) All successful essays become the property of the Association. (*c*) The medal shall be conferred, and honorable mention made of the two other essays considered worthy of this distinction, at a general meeting of the Association. (*d*) The competition is to be confined to those who at the time of entering the competition, as well as at the time of conferring the medal, shall be members of the American Medical Association. (*e*) The competition for the medal will be closed three months before the next annual meeting of the American Medical Association, and no essay will be received after March 1, 1898.

Competitors will address their essays to the undersigned.

J. McFADDEN GASTON, M. D., Chairman.

1½ Edgewood Ave., Atlanta,Ga.

—*Journal of the American Medical Association.*

